# Measurement data obtained by an instrumented steering wheel for driver model development

**DOI:** 10.1016/j.dib.2020.105485

**Published:** 2020-04-10

**Authors:** Massimiliano Gobbi, Francesco Comolli, Federico Maria Ballo, Gianpiero Mastinu

**Affiliations:** Department of Mechanical Engineering, Politecnico di Milano (Technical University), Via La Masa 1, 20156 Milan, Italy

**Keywords:** Steering wheel, ADAS, Automated vehicles, Driver model, Load cell

## Abstract

The data article presents the data acquired by an Instrumented Steering Wheel, able to measure the three force components and the three moment components applied by each of the two driver hands on an Instrumented Steering Wheel (ISW). Additionally, the ISW senses the grip forces at each hand.

In order to simulate emergency manoeuvres in a safe environment, a test track with a kick plate is used. Nine different drivers pass over the kick plate six times each. The drivers need to make an action on the steering wheel to counteract the lateral disturbance and recover the straight desired path.

The vehicle has been instrumented with an ISW and an inertial measuring unit. Data acquired by the two sensors have been synchronized and analysed. The force components due to mass properties of the ISW have been compensated in a proper way, to highlight the loads exerted only by the driver hands.

In the present data article, the data acquired during the described kick-plate test are reported for one driver during a single test. Discussion and conclusion have been presented in [1]. Data are provided in Matlab environment. Videos are provided to show how the manoeuvre occurs. The vehicle that was used for tests was modified with respect to the corresponding production vehicle. Data refer to the specific vehicle used in the tests that does not match with any vehicle produced by Toyota.

Specifications table

**Table:** 

Subject	Engineering
Specific subject area	Mechanical Engineering, automotive
Type of data	Figure Data
How data were acquired	Instrumented Steering Wheel by SMARTMechanical Company srl. Inertial measuring unit by OxTS.
Data format	Raw
Parameters for data collection	The data were acquired by using different sensors and synchronized. The data have been filtered and analysed, i.e. the fictitious (inertial) force components have been compensated.
Description of data collection	A vehicle was instrumented with an inertial measuring unit and an Instrumented Steering Wheel. Nine different driver pass over a kick plate, which suddenly shifts the rear axle of a vehicle which passes by. The vehicle straight motion is disturbed. The drivers act on the steering wheel to recover the desired path. The forces and moments applied on the ISW are measured and analysed. The raw data to validate the forces compensation of Fig. 6 are provided within the data article. The raw data of Fig. 7, acquired during a kick plate manoeuvre are provided within the data article.
Data source location	Institution: Centro Guida Sicura ACI-SARA di Lainate City/Town/Region: Lainate Country: Italy Latitude and longitude: 45°33′49.8″N 9°02′44.4″E
Data accessibility	With the article
Related research article	Authors: Massimiliano Gobbi, Francesco Comolli, Masatoshi Hada, Gianpiero Mastinu Title: An instrumented steering wheel for driver model development Journal: Mechatronics DOI: https://doi.org/10.1016/j.mechatronics.2019.102285

## Value of the Data

• The provided data improve the current knowledge of driver-vehicle interaction. Data refer to the specific vehicle used in the tests that does not match with any vehicle produced by Toyota.
• The provided data could be useful to study innovative driver models which consider the actual manner drivers apply forces and moments on the steering wheel.
• The provided data allow to develop new ADAS systems.
• According to Author's best knowledge, the provided data are the very first ones in the literature that describe accurately the forces and moments applied by each hand on the steering wheel by a driver
• The data are released for research purposes only. They are not intended for any use other than research and scientific investigations.

## 1 Data

**Fig. 1:**
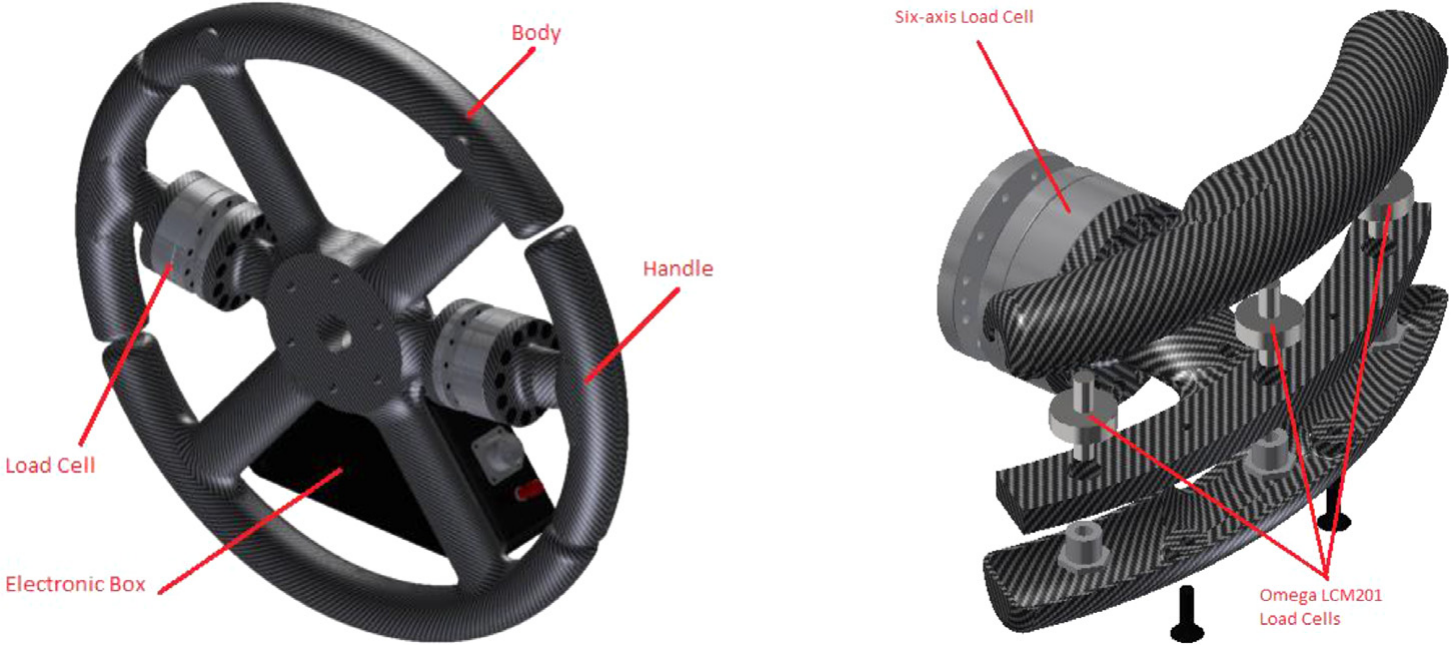
Instrumented Steering Wheel.

**Fig. 2:**
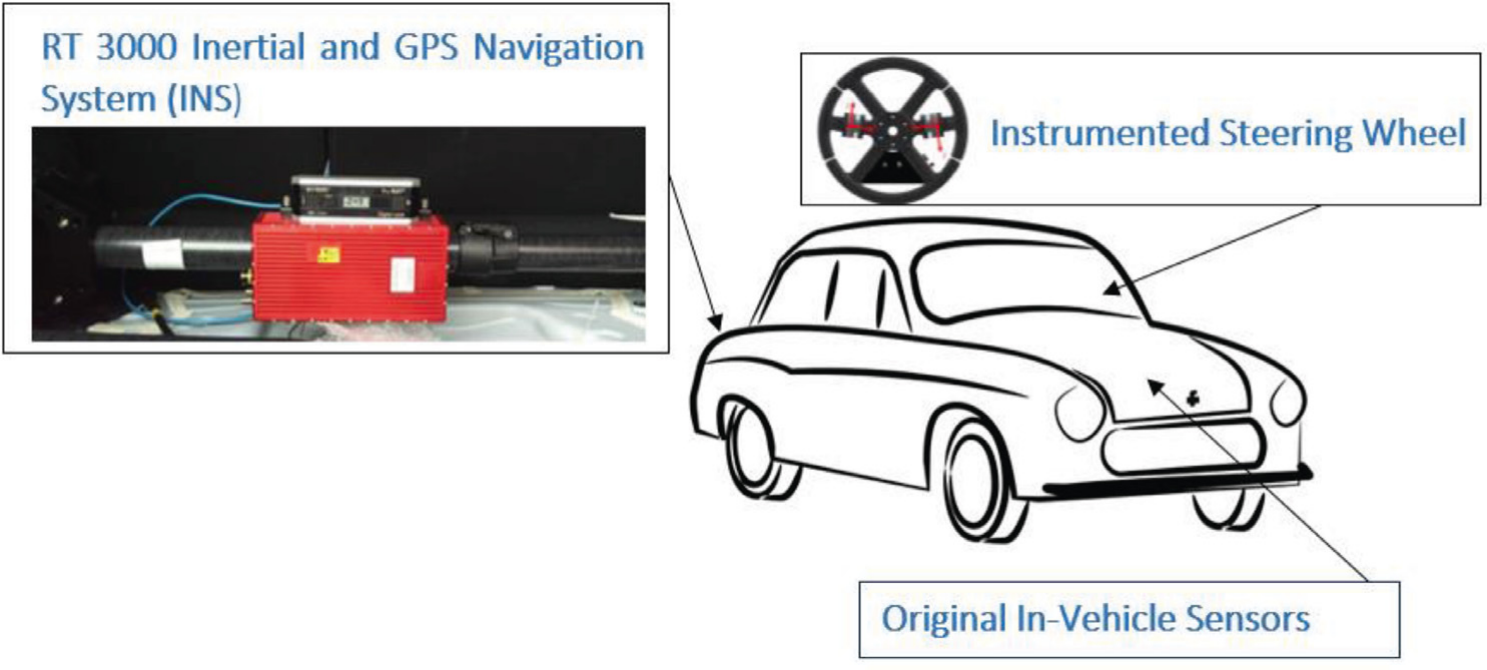
Sensors Adopted on the vehicle.

**Fig. 3:**
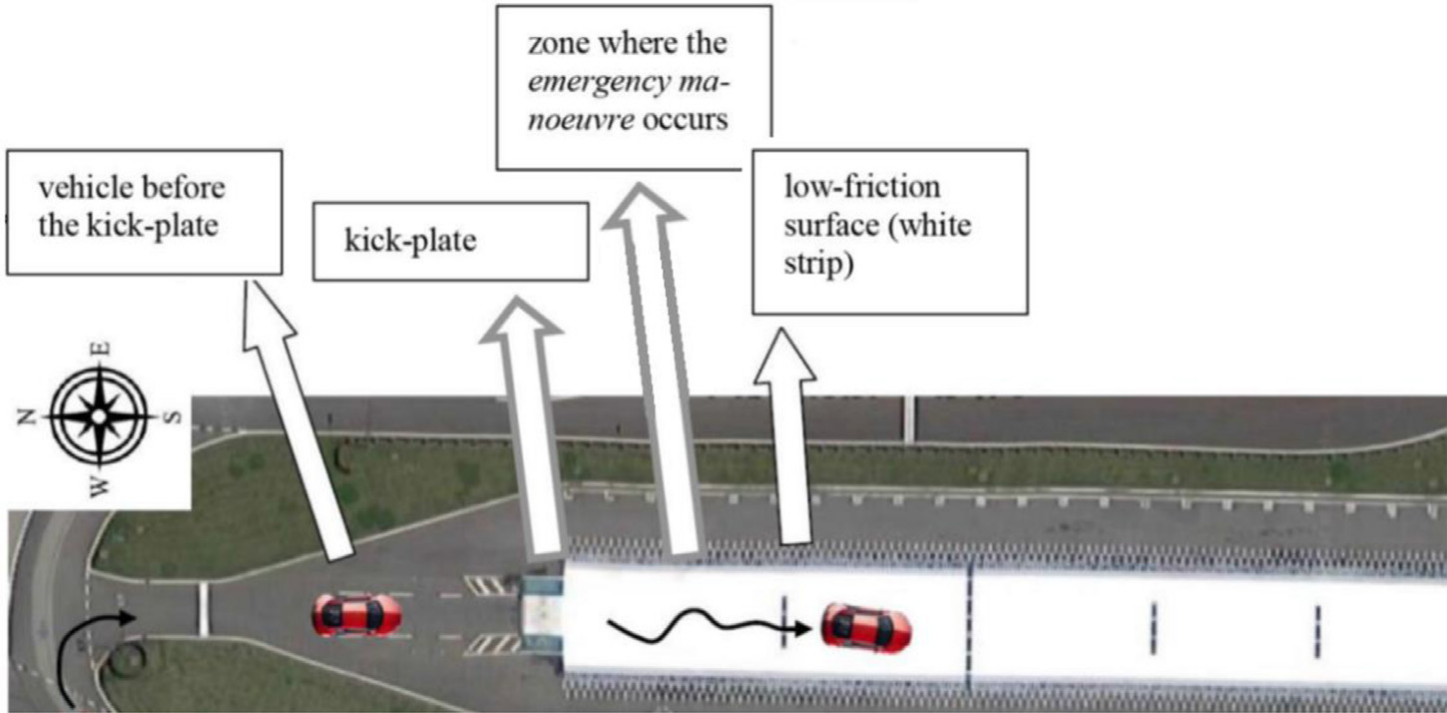
Kick plate plant in “Pista e Centro di Guida Sicura ACI-SARA di Lainate” [4].

**Fig. 4:**
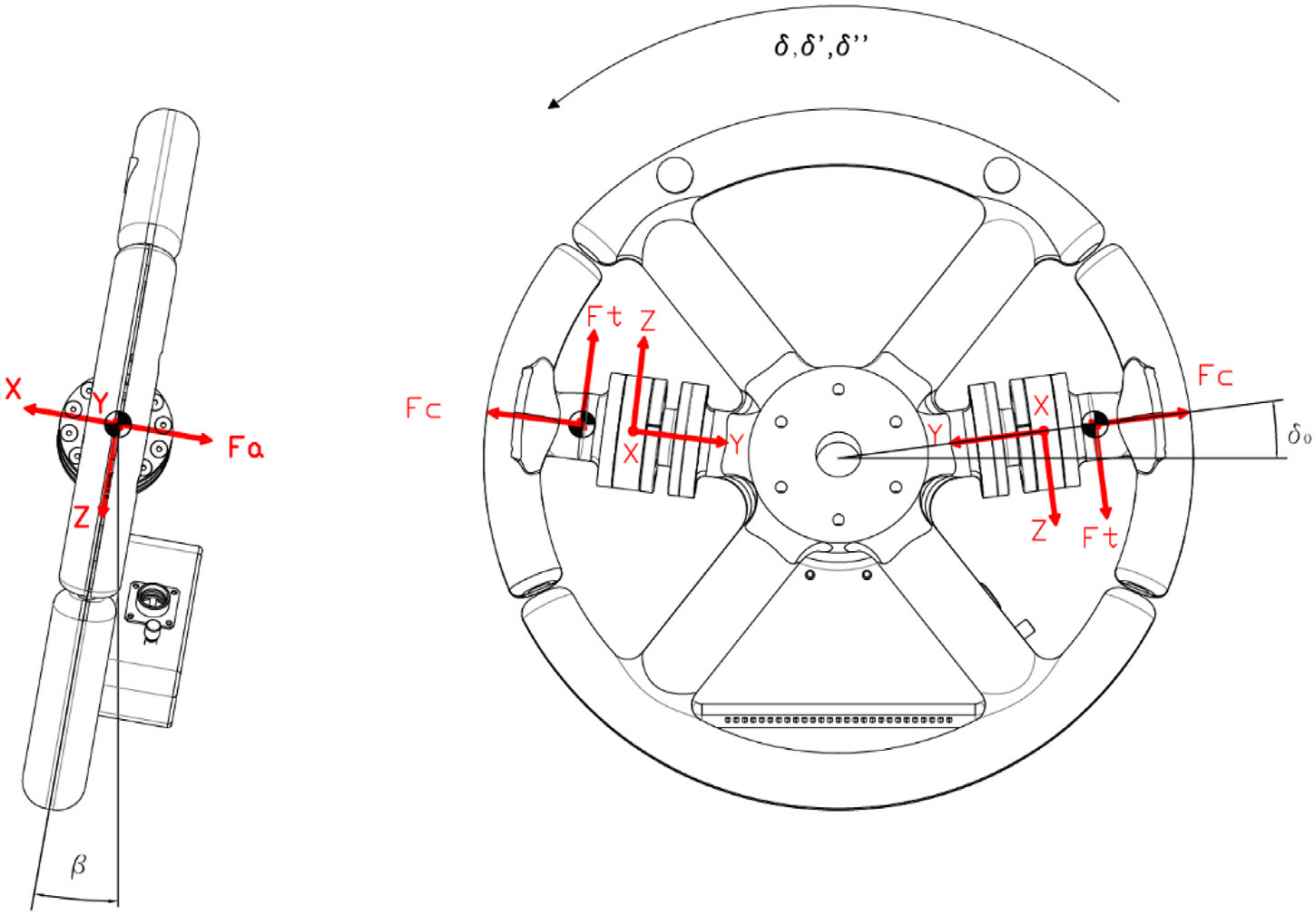
Force definitions.

**Fig. 5:**
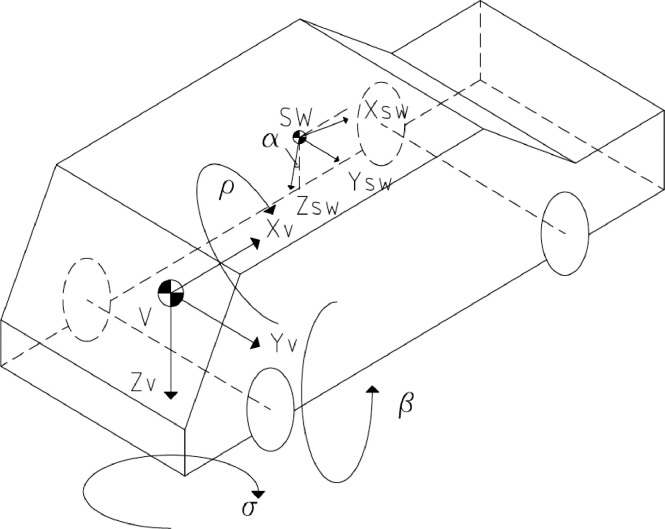
IMU and ISW reference frames.

**Fig. 6:**
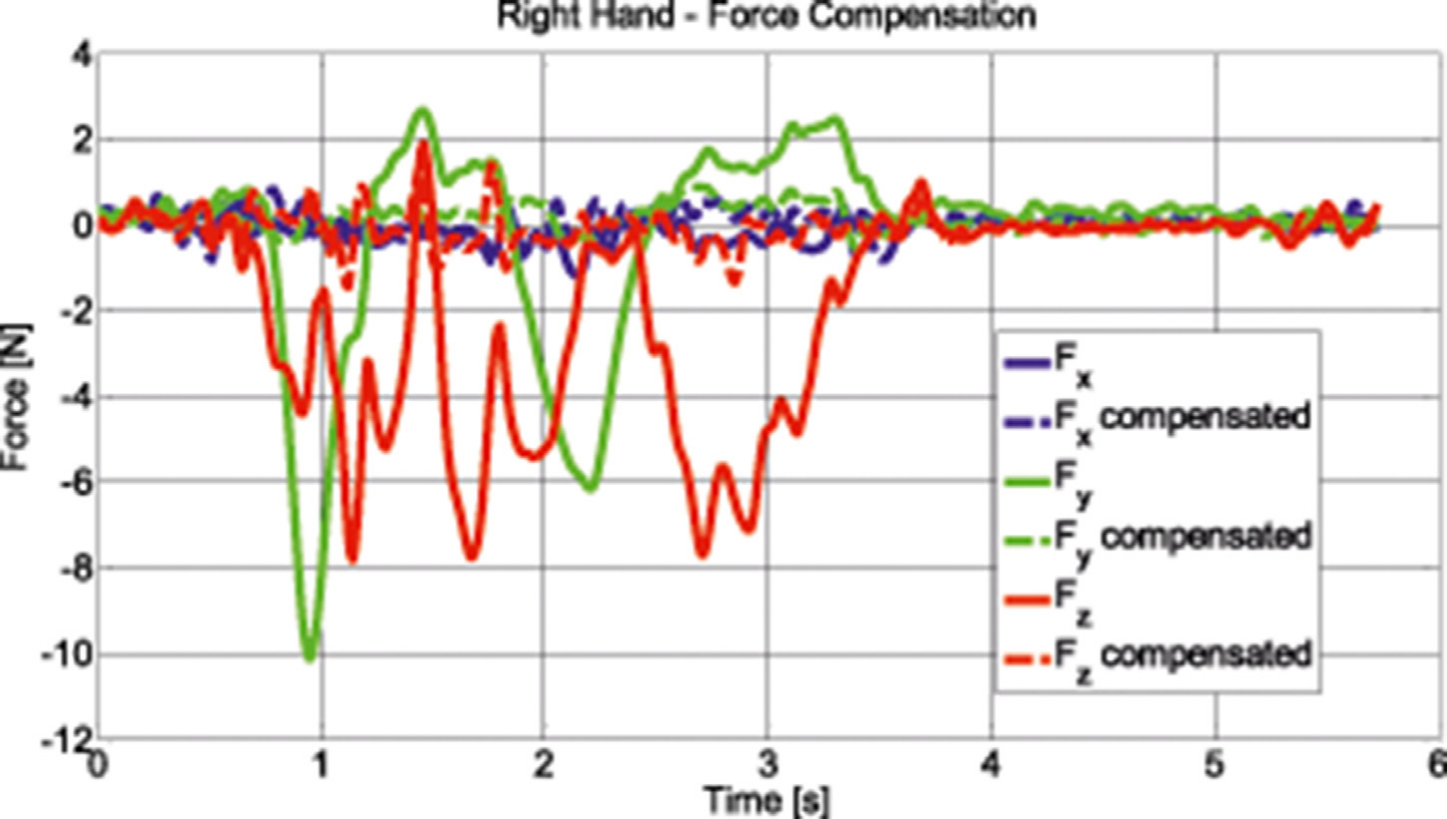
Forces compensation.

**Fig. 7:**
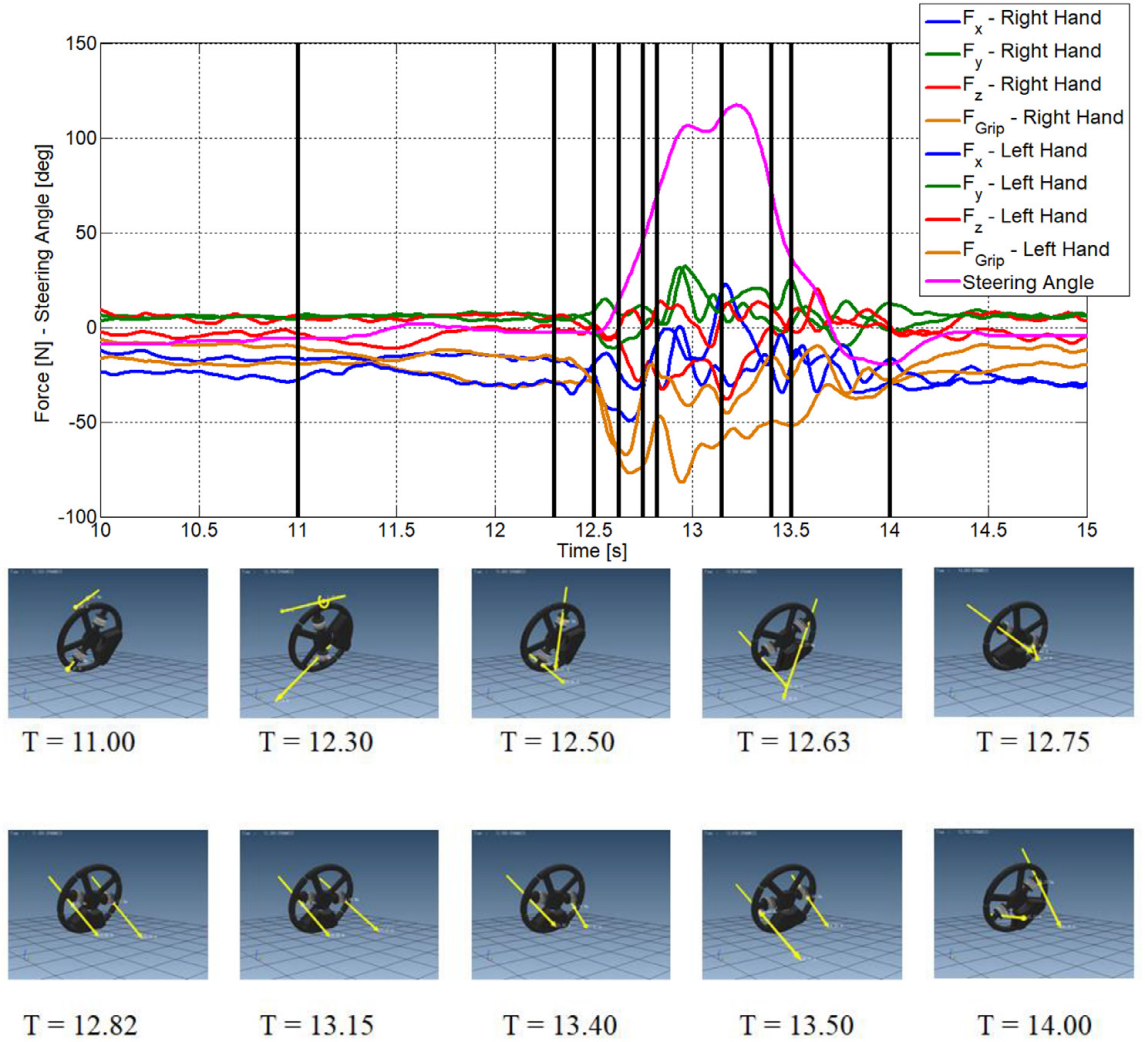
Forces acquired during a kick plate test by the ISW and video frames of the acquired force reconstruction.

Fig. 1 shows the Instrumented Steering Wheel, highlighting the main steering wheel components and the three mono-axial load cells for the grip force measurement. Fig. 2 describes the instrumentation installed on the test vehicle, the vehicle main specification is reported in Table 1. Fig. 3 describes the kick-plate manoeuvre which the drivers tackle in the test. Fig. 4 shows the forces which are measured by the Instrumented Steering Wheel due to vehicle motions. Fig. 5 shows the reference frames of the Inertial Measuring Unit (IMU) and of the Instrumented Steering Wheel (ISW). The raw data in raw_data_fig_6.txt are the forces exerted by the driver's right hand on the steering wheel during a kick plate test performed while keeping only the left hand on the steering wheel. The Matlab file plot_fig_6.m loads the data and plots Fig. 6, which shows the effect of the force compensation in a manoeuvre tackled keeping only one hand on the steering wheel. The raw data in raw_data_fig_7.txt are the forces and the moments exerted by a driver on the steering wheel during a kick plate test. The Matlab file plot_fig_7.m loads the data and plots Fig. 7 which shows the forces acquired during a kick-plate manoeuvre and shows video frames of the acquired total force. The video Forces.wmv contains the resultant forces applied at the ISW during a kick plate manoeuvre, from which are taken the video frames in Fig. 7. The video Kickplate.wmv shows the vehicle tackling the kick plate manoeuvre, recorded by an external viewer. We provided the data of five drivers, each one passing over the kick plate three times, in order to guarantee statistical significance of the data.

**Table 1:** Test Vehicle Specification.

Loaded vehicle mass	1740	kg
Weight Distribution front-rear axle	52.5–47.5%	
Wheelbase	2620	mm
Tread	1525	mm
Steering gear ratio	15	
Tyres	225/45 R17	
CoG Height a	562.5	mm
Yaw moment of inertia (loaded vehicle)*	2600	kgm^2^

a Estimates using the formula presented in [2].

## 2 Experimental design, materials, and methods

An Instrumented Steering Wheel (ISW) able to measure the three force components and the three moment components exerted by each driver hand is used. The ISW measures the forces and moments applied at two handles by using two six-axis load cells (Fig. 1). In addition, the ISW measures the grip force (the force by which the driver holds firmly the steering wheel). Such grip force is sensed by means of three mono-axial load cells at each handle [1].

In addition to the ISW, the vehicle was equipped with an inertial measuring unit OxTS RT3000 in the vehicle trunk (Fig. 2). Such system was used to derive the vehicle motion state variables. Vehicle dynamics data were needed for the compensation of forces and moments at the ISW. In fact, due to mass properties of the handles of the ISW, Coriolis and other inertia effects are sensed by the ISW. Since we are interested in the forces and moments applied by the driver only, then such fictitious (inertial) forces had to be removed. To derive the position of the vehicle in the track, two GPS antennas were fitted on the roof and a GPS beacon was positioned on the track to increase the GPS accuracy. Finally, the sensors embedded in the vehicle were acquired via the vehicle CAN (Controller Area Network) to measure the steering wheel angle and its velocity (Fig. 2). The steering wheel is power supplied by means of the original clock spring cable, and the signals are sent via the clock spring to a CAN bus interface (Kvaser USB CAN Pro 5xHS) which acquires the data from the ISW CAN and from the vehicle CAN. Those data are saved on a laptop, together with the data acquired from the inertial measuring unit (which manages the GPS signal). The vehicle specification is reported in Table 1.

The track “Pista e Centro di Guida Sicura ACI-SARA di Lainate” is equipped with a kick-plate, which is used to simulate emergency manoeuvres in a safe environment. The test vehicle goes straight ahead at a constant speed of 40 [km/h]. When the rear axle passes over the kick-plate, the plate is displaced laterally imposing a lateral disturbance to the vehicle straight trajectory, simulating a hard disturbance. To recover the straight motion, a fast counter-steering is required. Nine different drivers, with different driving experience, performed the manoeuvre six times each [3] [4]. The disturbance direction was random, while the disturbance intensity was set on a low level for the first three passages, on a higher level for the other three passages.

The forces and moments measured by the ISW are the sum of the loads applied by the driver ${\vec{F}}_{applied}$, the weight components of the ISW handles ${\vec{F}}_{weight}$, the forces due to the inertia of the handles subjected to the vehicle accelerations ${\vec{F}}_{inertial}$, and the centrifugal and tangential (Coriolis) forces due to the high rate steering rotation ${\vec{F}}_{rotation}$.

(1) $${\vec{F}}_{applied}={\vec{F}}_{measured}-{\vec{F}}_{weight}-{\vec{F}}_{inertial}-{\vec{F}}_{rotation}$$

The weight components are functions of the handles masses, the steering angle and the inclination angle of the ISW with respect to the vertical direction (Fig. 4).

(2) $${\vec{F}}_{weight}=\Lambda_{weight}\left(\delta ,\beta \right)\cdot m_{H}\cdot g$$

The high rate rotation components are functions of the steering angle rate and acceleration.

(3) $${\vec{F}}_{rotation}=-\;m\underset{-}{\ddot{\delta }}\;\wedge \underset{-}{r_{h}}-\;m\underset{-}{\ddot{\delta }}\;\;\wedge \left(\underset{-}{\dot{\delta }}\wedge \underset{-}{r_{h}}\right)$$

The inertial components are functions of the vehicle accelerations measured by the inertial measuring unit in the trunk, reported to the load cells reference frames (Fig. 5).

(4) $${\vec{F}}_{intertial}=-m_{H}{\vec{a}}_{v}=-\;m\;\Lambda_{SW-LC}\;\Lambda_{RT-SW}\underset{-}{\ddot{q}}$$

The Coriolis components have been neglected because of the small magnitudes.

In Fig. 6 it is possible to see the forces compensation during a kick plate test performed keeping only one hand on the steering wheel. In such a way, vanishing forces and moments are expected on one of the Load Cells. The solid line in Fig. 6 represents the measured forces, which are not null. The dashed line represents the forces compensated as shown.

Finally, the grip force *F_grip_* is computed by the three contributions of the mono-axial load cells in each handles by Eq. (5), where $F_{x_{six-axis\;load\;cell}}$ is the load force measured by the six-axis load cell in the axial direction and *F_i_* is the load force measured by each of the handle load cells. The handle load cells measure only the force in the axial direction of the ISW.

(5) $$F_{grip}=\min\left(|F_{x_{six-axis\;load\;cell}}-\sum_{i=1}^{3}F_{i}|,\;|\sum_{i=1}^{3}F_{i}|\right)$$

In Fig. 7 the force components acquired by the ISW on a kick plate test are shown. Here the axial (*F_x_*), radial (*F_y_*) and tangential (*F_z_*) forces for each hand are reported, in addition with the grip forces *F_grip_* (please refer to Fig. 4 for the force directions). In Fig. 7 the resultant forces in specific time instants are shown. Forces that are depicted in Fig. 7 do take into account the moments applied by the driver's hands.

We have provided two “.txt” data files that contain the data described above, both Fig. 6 and Fig. 7. The structure of the database is described in the executable Matlab files. We have provided “.m” executable Matlab files that processes the data in the “.txt” files and return plots.

We have also provided a video to show how the forces do vary on the steering wheel during an emergency manoeuvre.

We have provided a video of the car we used while passing by the kick-plate.

The vehicle that was used for tests was modified with respect to the corresponding production vehicle. Due to such modifications the vehicle used for tests does not correspond to any vehicle running on normal roads.

## Declaration of Competing Interest

The authors declare that they have no known competing financial interests or personal relationships that could have appeared to influence the work reported in this paper.

## Appendix Supplementary materials

Image, application 1

Image, application 2
